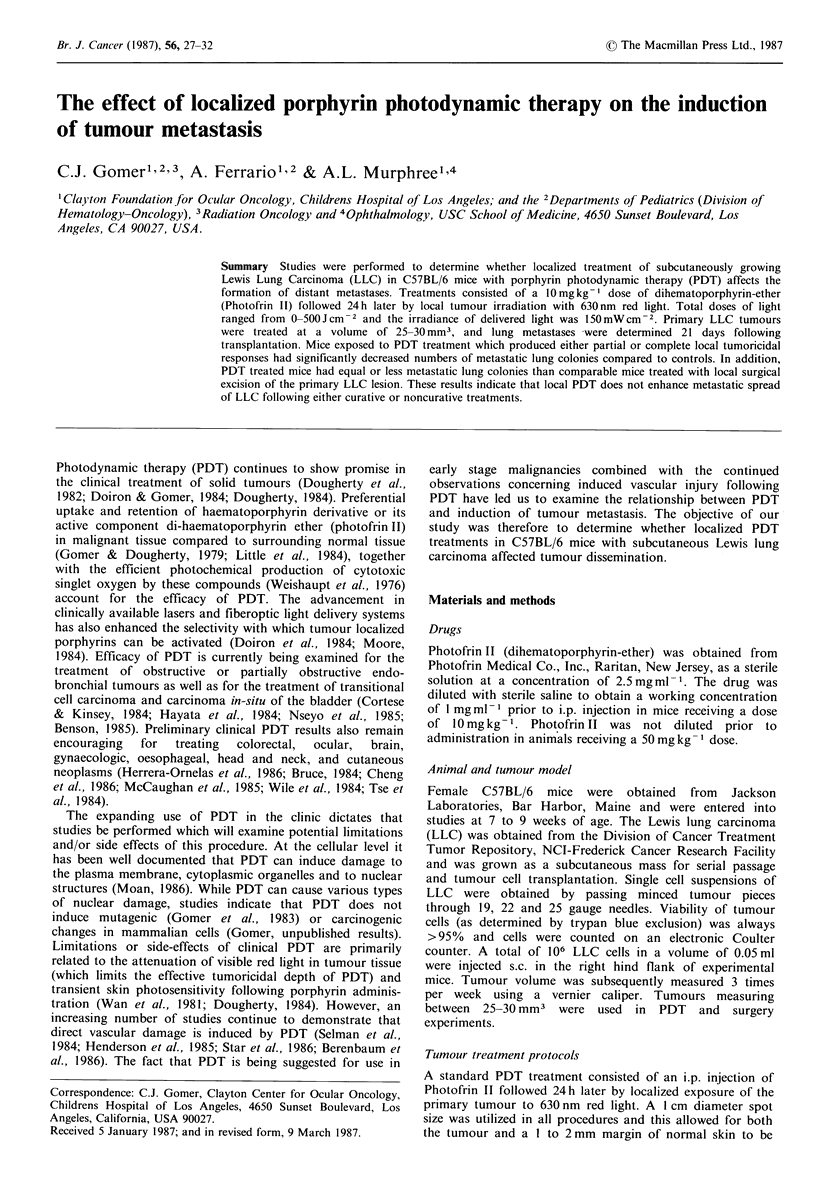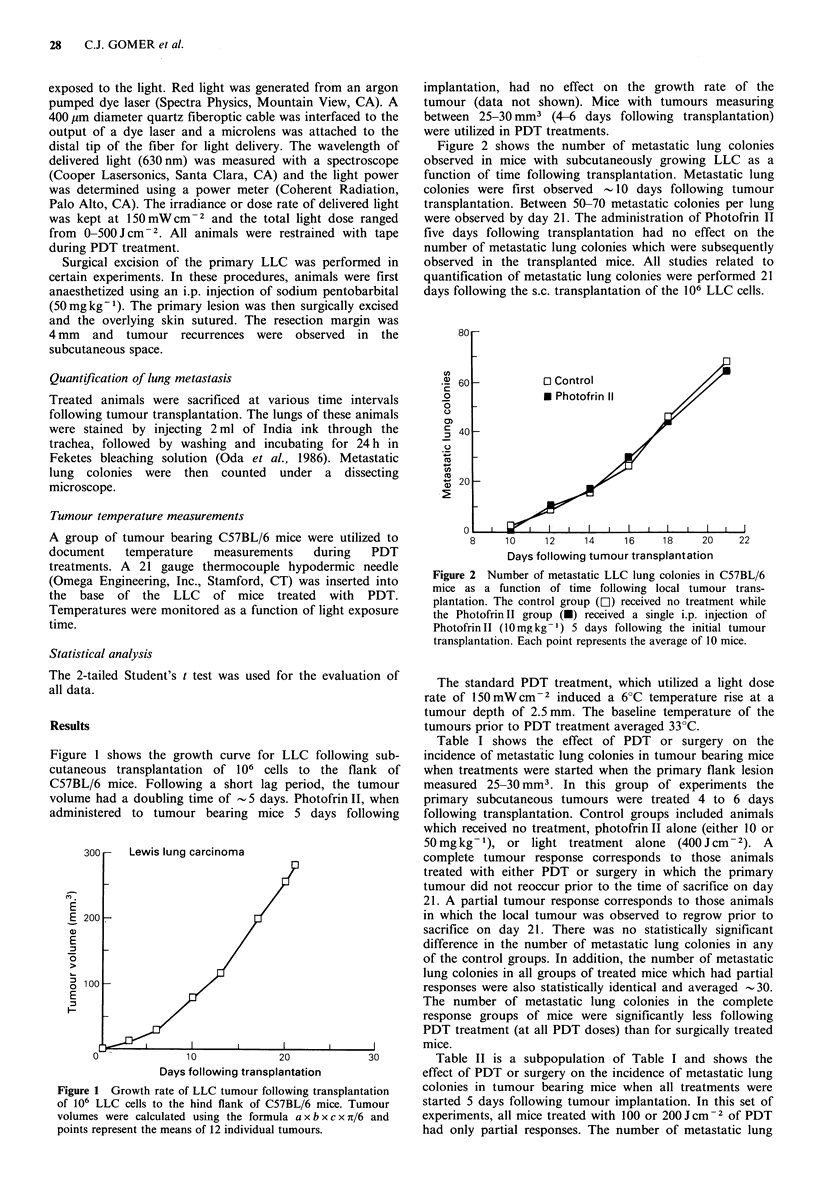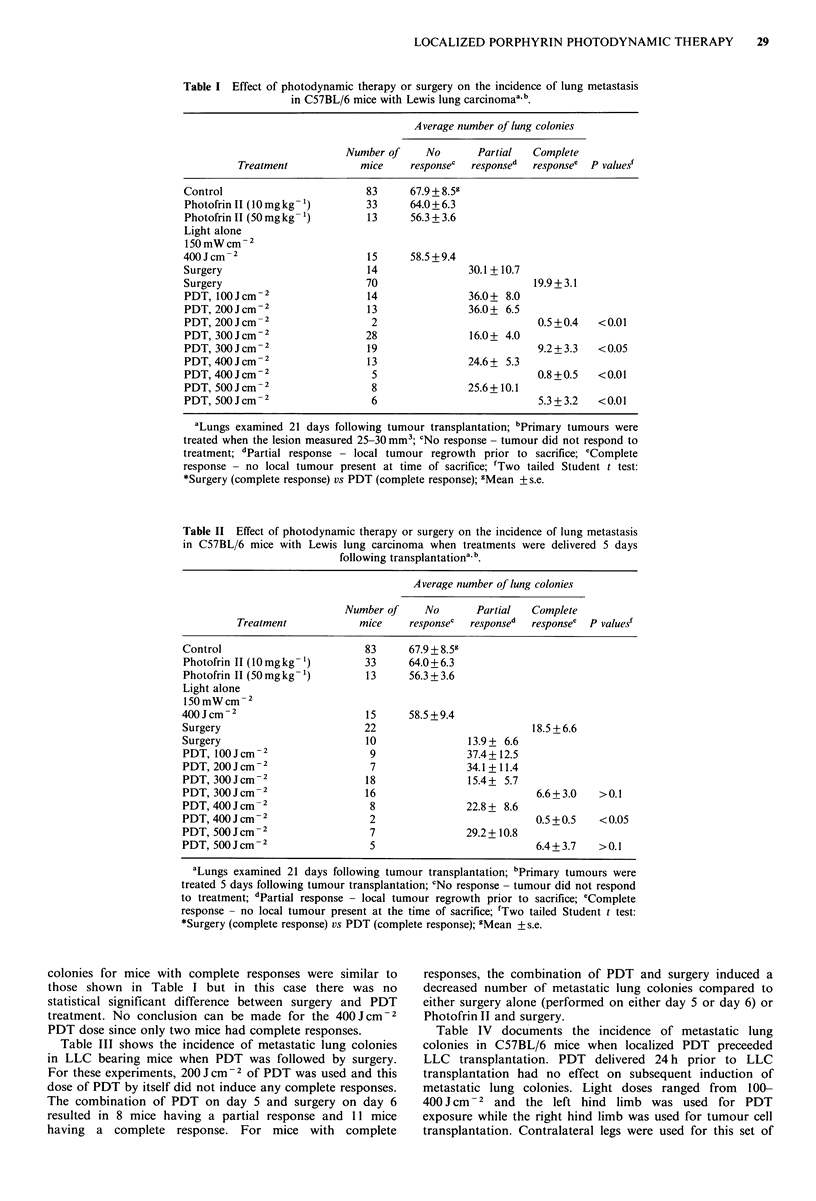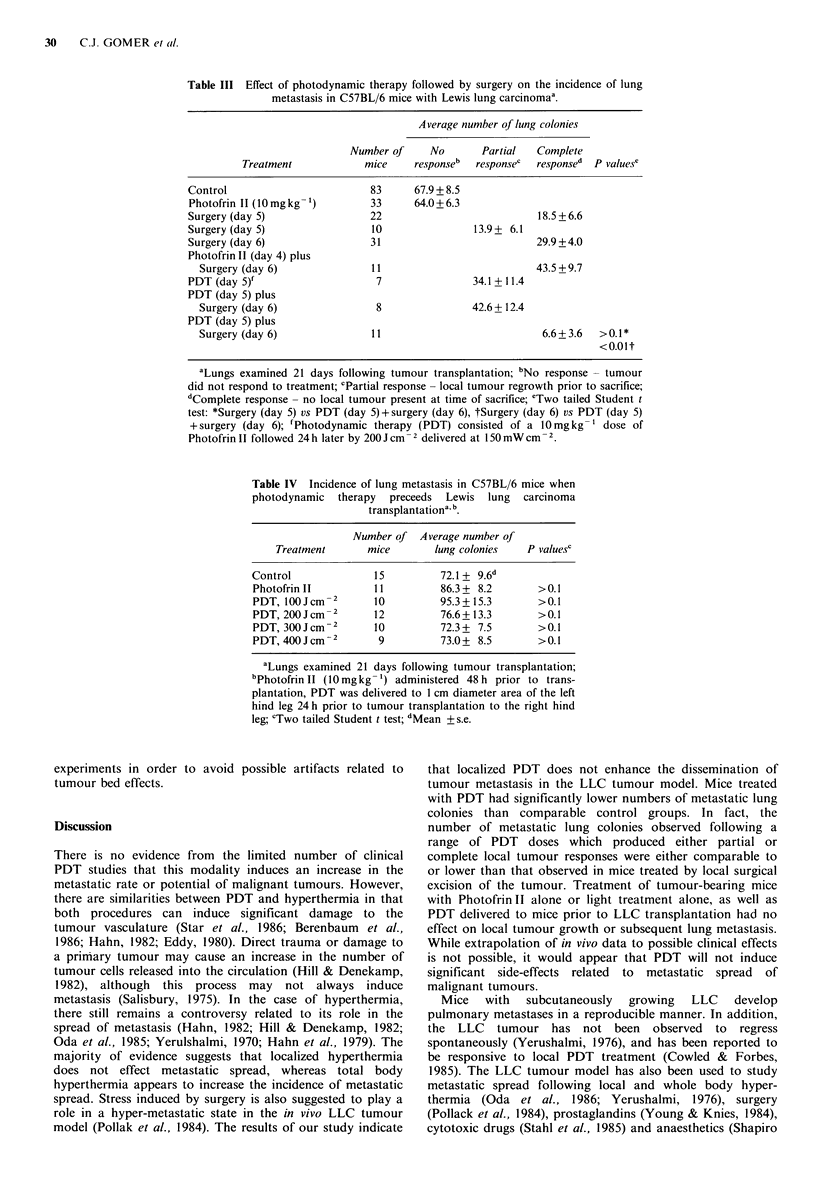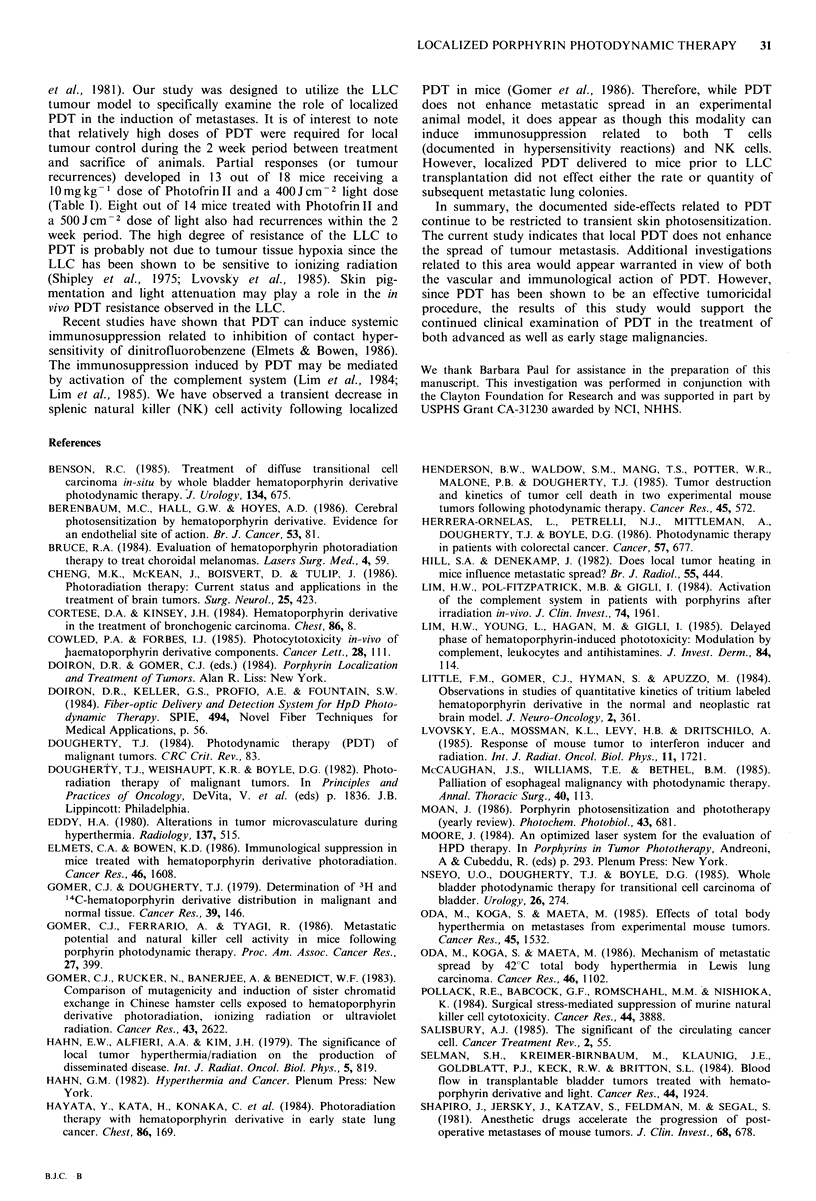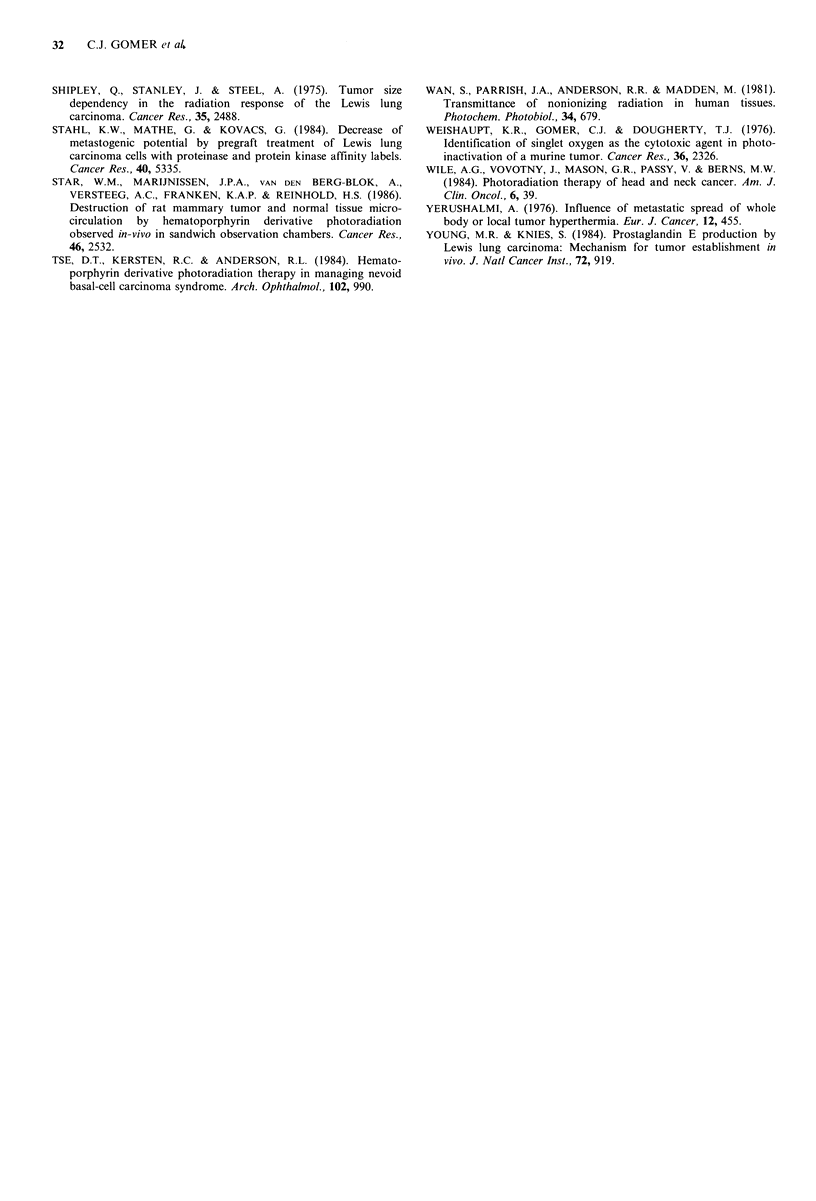# The effect of localized porphyrin photodynamic therapy on the induction of tumour metastasis.

**DOI:** 10.1038/bjc.1987.147

**Published:** 1987-07

**Authors:** C. J. Gomer, A. Ferrario, A. L. Murphree

## Abstract

Studies were performed to determine whether localized treatment of subcutaneously growing Lewis Lung Carcinoma (LLC) in C57BL/6 mice with porphyrin photodynamic therapy (PDT) affects the formation of distant metastases. Treatments consisted of a 10 mg kg-1 dose of dihematoporphyrin-ether (Photofrin II) followed 24 h later by local tumour irradiation with 630 nm red light. Total doses of light ranged from 0-500 J cm-2 and the irradiance of delivered light was 150 mW cm-2. Primary LLC tumours were treated at a volume of 25-30 mm3, and lung metastases were determined 21 days following transplantation. Mice exposed to PDT treatment which produced either partial or complete local tumoricidal responses had significantly decreased numbers of metastatic lung colonies compared to controls. In addition, PDT treated mice had equal or less metastatic lung colonies than comparable mice treated with local surgical excision of the primary LLC lesion. These results indicate that local PDT does not enhance metastatic spread of LLC following either curative or noncurative treatments.


Br. J. Cancer (1987), 56, 27–32 © The Macmillan Press Ltd., 1987

### The effect of localized porphyrin photodynamic therapy on the induction of tumour metastasis

C.J. Gomer[1,2,3], A. Ferrario[1,2] & A.L. Murphree[1,4]

[1]*Clayton Foundation for Ocular Oncology, Childrens Hospital of Los Angeles; and the* [2]*Departments of Pediatrics (Division of Hematology–Oncology),* [3]*Radiation Oncology and* [4]*Ophthalmology, USC School of Medicine, 4650 Sunset Boulevard, Los Angeles, CA 90027, USA.*

**Summary** Studies were performed to determine whether localized treatment of subcutaneously growing Lewis Lung Carcinoma (LLC) in C57BL/6 mice with porphyrin photodynamic therapy (PDT) affects the formation of distant metastases. Treatments consisted of a $10\,\mathrm{mg\,kg^{-1}}$ dose of dihematoporphyrin-ether (Photofrin II) followed 24h later by local tumour irradiation with 630nm red light. Total doses of light ranged from $0–500\,\mathrm{J\,cm^{-2}}$ and the irradiance of delivered light was $150\,\mathrm{mW\,cm^{-2}}$. Primary LLC tumours were treated at a volume of $25–30\,\mathrm{mm^3}$, and lung metastases were determined 21 days following transplantation. Mice exposed to PDT treatment which produced either partial or complete local tumoricidal responses had significantly decreased numbers of metastatic lung colonies compared to controls. In addition, PDT treated mice had equal or less metastatic lung colonies than comparable mice treated with local surgical excision of the primary LLC lesion. These results indicate that local PDT does not enhance metastatic spread of LLC following either curative or noncurative treatments.

Photodynamic therapy (PDT) continues to show promise in the clinical treatment of solid tumours (Dougherty *et al.*, 1982; Doiron & Gomer, 1984; Dougherty, 1984). Preferential uptake and retention of haematoporphyrin derivative or its active component di-haematoporphyrin ether (photofrin II) in malignant tissue compared to surrounding normal tissue (Gomer & Dougherty, 1979; Little *et al.*, 1984), together with the efficient photochemical production of cytotoxic singlet oxygen by these compounds (Weishaupt *et al.*, 1976) account for the efficacy of PDT. The advancement in clinically available lasers and fiberoptic light delivery systems has also enhanced the selectivity with which tumour localized porphyrins can be activated (Doiron *et al.*, 1984; Moore, 1984). Efficacy of PDT is currently being examined for the treatment of obstructive or partially obstructive endobronchial tumours as well as for the treatment of transitional cell carcinoma and carcinoma *in-situ* of the bladder (Cortese & Kinsey, 1984; Hayata *et al.*, 1984; Nseyo *et al.*, 1985; Benson, 1985). Preliminary clinical PDT results also remain encouraging for treating colorectal, ocular, brain, gynaecologic, oesophageal, head and neck, and cutaneous neoplasms (Herrera-Ornelas *et al.*, 1986; Bruce, 1984; Cheng *et al.*, 1986; McCaughan *et al.*, 1985; Wile *et al.*, 1984; Tse *et al.*, 1984).

The expanding use of PDT in the clinic dictates that studies be performed which will examine potential limitations and/or side effects of this procedure. At the cellular level it has been well documented that PDT can induce damage to the plasma membrane, cytoplasmic organelles and to nuclear structures (Moan, 1986). While PDT can cause various types of nuclear damage, studies indicate that PDT does not induce mutagenic (Gomer *et al.*, 1983) or carcinogenic changes in mammalian cells (Gomer, unpublished results). Limitations or side-effects of clinical PDT are primarily related to the attenuation of visible red light in tumour tissue (which limits the effective tumoricidal depth of PDT) and transient skin photosensitivity following porphyrin administration (Wan *et al.*, 1981; Dougherty, 1984). However, an increasing number of studies continue to demonstrate that direct vascular damage is induced by PDT (Selman *et al.*, 1984; Henderson *et al.*, 1985; Star *et al.*, 1986; Berenbaum *et al.*, 1986). The fact that PDT is being suggested for use in early stage malignancies combined with the continued observations concerning induced vascular injury following PDT have led us to examine the relationship between PDT and induction of tumour metastasis. The objective of our study was therefore to determine whether localized PDT treatments in C57BL/6 mice with subcutaneous Lewis lung carcinoma affected tumour dissemination.

Correspondence: C.J. Gomer, Clayton Center for Ocular Oncology, Childrens Hospital of Los Angeles, 4650 Sunset Boulevard, Los Angeles, California, USA 90027.
Received 5 January 1987; and in revised form, 9 March 1987.

### Materials and methods

### *Drugs*

Photofrin II (dihematoporphyrin-ether) was obtained from Photofrin Medical Co., Inc., Raritan, New Jersey, as a sterile solution at a concentration of $2.5\,\mathrm{mg\,ml^{-1}}$. The drug was diluted with sterile saline to obtain a working concentration of $1\,\mathrm{mg\,ml^{-1}}$ prior to i.p. injection in mice receiving a dose of $10\,\mathrm{mg\,kg^{-1}}$. Photofrin II was not diluted prior to administration in animals receiving a $50\,\mathrm{mg\,kg^{-1}}$ dose.

### *Animal and tumour model*

Female C57BL/6 mice were obtained from Jackson Laboratories, Bar Harbor, Maine and were entered into studies at 7 to 9 weeks of age. The Lewis lung carcinoma (LLC) was obtained from the Division of Cancer Treatment Tumor Repository, NCI-Frederick Cancer Research Facility and was grown as a subcutaneous mass for serial passage and tumour cell transplantation. Single cell suspensions of LLC were obtained by passing minced tumour pieces through 19, 22 and 25 gauge needles. Viability of tumour cells (as determined by trypan blue exclusion) was always >95% and cells were counted on an electronic Coulter counter. A total of $10^6$ LLC cells in a volume of 0.05 ml were injected s.c. in the right hind flank of experimental mice. Tumour volume was subsequently measured 3 times per week using a vernier caliper. Tumours measuring between $25–30\,\mathrm{mm^3}$ were used in PDT and surgery experiments.

### *Tumour treatment protocols*

A standard PDT treatment consisted of an i.p. injection of Photofrin II followed 24h later by localized exposure of the primary tumour to 630nm red light. A 1 cm diameter spot size was utilized in all procedures and this allowed for both the tumour and a 1 to 2 mm margin of normal skin to be exposed to the light. Red light was generated from an argon pumped dye laser (Spectra Physics, Mountain View, CA). A 400 $\mu$m diameter quartz fiberoptic cable was interfaced to the output of a dye laser and a microlens was attached to the distal tip of the fiber for light delivery. The wavelength of delivered light (630 nm) was measured with a spectroscope (Cooper Lasersonics, Santa Clara, CA) and the light power was determined using a power meter (Coherent Radiation, Palo Alto, CA). The irradiance or dose rate of delivered light was kept at 150 mW cm$^{-2}$ and the total light dose ranged from 0–500 J cm$^{-2}$. All animals were restrained with tape during PDT treatment.

Surgical excision of the primary LLC was performed in certain experiments. In these procedures, animals were first anaesthetized using an i.p. injection of sodium pentobarbital (50 mg kg$^{-1}$). The primary lesion was then surgically excised and the overlying skin sutured. The resection margin was 4 mm and tumour recurrences were observed in the subcutaneous space.

*Quantification of lung metastasis*

Treated animals were sacrificed at various time intervals following tumour transplantation. The lungs of these animals were stained by injecting 2 ml of India ink through the trachea, followed by washing and incubating for 24 h in Feketes bleaching solution (Oda *et al.*, 1986). Metastatic lung colonies were then counted under a dissecting microscope.

*Tumour temperature measurements*

A group of tumour bearing C57BL/6 mice were utilized to document temperature measurements during PDT treatments. A 21 gauge thermocouple hypodermic needle (Omega Engineering, Inc., Stamford, CT) was inserted into the base of the LLC of mice treated with PDT. Temperatures were monitored as a function of light exposure time.

*Statistical analysis*

The 2-tailed Student's $t$ test was used for the evaluation of all data.

### Results

Figure 1 shows the growth curve for LLC following subcutaneous transplantation of $10^6$ cells to the flank of C57BL/6 mice. Following a short lag period, the tumour volume had a doubling time of ~5 days. Photofrin II, when administered to tumour bearing mice 5 days following

**Figure 1** Growth rate of LLC tumour following transplantation of $10^6$ LLC cells to the hind flank of C57BL/6 mice. Tumour volumes were calculated using the formula $a \times b \times c \times \pi/6$ and points represent the means of 12 individual tumours.

implantation, had no effect on the growth rate of the tumour (data not shown). Mice with tumours measuring between 25–30 mm$^3$ (4–6 days following transplantation) were utilized in PDT treatments.

Figure 2 shows the number of metastatic lung colonies observed in mice with subcutaneously growing LLC as a function of time following transplantation. Metastatic lung colonies were first observed ~10 days following tumour transplantation. Between 50–70 metastatic colonies per lung were observed by day 21. The administration of Photofrin II five days following transplantation had no effect on the number of metastatic lung colonies which were subsequently observed in the transplanted mice. All studies related to quantification of metastatic lung colonies were performed 21 days following the s.c. transplantation of the $10^6$ LLC cells.

**Figure 2** Number of metastatic LLC lung colonies in C57BL/6 mice as a function of time following local tumour transplantation. The control group (□) received no treatment while the Photofrin II group (■) received a single i.p. injection of Photofrin II (10 mg kg$^{-1}$) 5 days following the initial tumour transplantation. Each point represents the average of 10 mice.

The standard PDT treatment, which utilized a light dose rate of 150 mW cm$^{-2}$ induced a 6°C temperature rise at a tumour depth of 2.5 mm. The baseline temperature of the tumours prior to PDT treatment averaged 33°C.

Table I shows the effect of PDT or surgery on the incidence of metastatic lung colonies in tumour bearing mice when treatments were started when the primary flank lesion measured 25–30 mm$^3$. In this group of experiments the primary subcutaneous tumours were treated 4 to 6 days following transplantation. Control groups included animals which received no treatment, photofrin II alone (either 10 or 50 mg kg$^{-1}$), or light treatment alone (400 J cm$^{-2}$). A complete tumour response corresponds to those animals treated with either PDT or surgery in which the primary tumour did not reoccur prior to the time of sacrifice on day 21. A partial tumour response corresponds to those animals in which the local tumour was observed to regrow prior to sacrifice on day 21. There was no statistically significant difference in the number of metastatic lung colonies in any of the control groups. In addition, the number of metastatic lung colonies in all groups of treated mice which had partial responses were also statistically identical and averaged ~30. The number of metastatic lung colonies in the complete response groups of mice were significantly less following PDT treatment (at all PDT doses) than for surgically treated mice.

Table II is a subpopulation of Table I and shows the effect of PDT or surgery on the incidence of metastatic lung colonies in tumour bearing mice when all treatments were started 5 days following tumour implantation. In this set of experiments, all mice treated with 100 or 200 J cm$^{-2}$ of PDT had only partial responses. The number of metastatic lung

**Table I** Effect of photodynamic therapy or surgery on the incidence of lung metastasis in C57BL/6 mice with Lewis lung carcinoma[a,b].

| Treatment | Number of mice | *Average number of lung colonies* No response[c] | Partial response[d] | Complete response[e] | P values[f] |
|---|---|---|---|---|---|
| Control | 83 | 67.9±8.5[g] | | | |
| Photofrin II (10 mg kg$^{-1}$) | 33 | 64.0±6.3 | | | |
| Photofrin II (50 mg kg$^{-1}$) | 13 | 56.3±3.6 | | | |
| Light alone | | | | | |
| 150 mW cm$^{-2}$ | | | | | |
| 400 J cm$^{-2}$ | 15 | 58.5±9.4 | | | |
| Surgery | 14 | | 30.1±10.7 | | |
| Surgery | 70 | | | 19.9±3.1 | |
| PDT, 100 J cm$^{-2}$ | 14 | | 36.0± 8.0 | | |
| PDT, 200 J cm$^{-2}$ | 13 | | 36.0± 6.5 | | |
| PDT, 200 J cm$^{-2}$ | 2 | | | 0.5±0.4 | <0.01 |
| PDT, 300 J cm$^{-2}$ | 28 | | 16.0± 4.0 | | |
| PDT, 300 J cm$^{-2}$ | 19 | | | 9.2±3.3 | <0.05 |
| PDT, 400 J cm$^{-2}$ | 13 | | 24.6± 5.3 | | |
| PDT, 400 J cm$^{-2}$ | 5 | | | 0.8±0.5 | <0.01 |
| PDT, 500 J cm$^{-2}$ | 8 | | 25.6±10.1 | | |
| PDT, 500 J cm$^{-2}$ | 6 | | | 5.3±3.2 | <0.01 |

[a]Lungs examined 21 days following tumour transplantation; [b]Primary tumours were treated when the lesion measured 25–30 mm$^3$; [c]No response – tumour did not respond to treatment; [d]Partial response – local tumour regrowth prior to sacrifice; [e]Complete response – no local tumour present at time of sacrifice; [f]Two tailed Student *t* test: *Surgery (complete response) *vs* PDT (complete response); [g]Mean ±s.e.

**Table II** Effect of photodynamic therapy or surgery on the incidence of lung metastasis in C57BL/6 mice with Lewis lung carcinoma when treatments were delivered 5 days following transplantation[a,b].

| Treatment | Number of mice | *Average number of lung colonies* No response[c] | Partial response[d] | Complete response[e] | P values[f] |
|---|---|---|---|---|---|
| Control | 83 | 67.9±8.5[g] | | | |
| Photofrin II (10 mg kg$^{-1}$) | 33 | 64.0±6.3 | | | |
| Photofrin II (50 mg kg$^{-1}$) | 13 | 56.3±3.6 | | | |
| Light alone | | | | | |
| 150 mW cm$^{-2}$ | | | | | |
| 400 J cm$^{-2}$ | 15 | 58.5±9.4 | | | |
| Surgery | 22 | | | 18.5±6.6 | |
| Surgery | 10 | | 13.9± 6.6 | | |
| PDT, 100 J cm$^{-2}$ | 9 | | 37.4±12.5 | | |
| PDT, 200 J cm$^{-2}$ | 7 | | 34.1±11.4 | | |
| PDT, 300 J cm$^{-2}$ | 18 | | 15.4± 5.7 | | |
| PDT, 300 J cm$^{-2}$ | 16 | | | 6.6±3.0 | >0.1 |
| PDT, 400 J cm$^{-2}$ | 8 | | 22.8± 8.6 | | |
| PDT, 400 J cm$^{-2}$ | 2 | | | 0.5±0.5 | <0.05 |
| PDT, 500 J cm$^{-2}$ | 7 | | 29.2±10.8 | | |
| PDT, 500 J cm$^{-2}$ | 5 | | | 6.4±3.7 | >0.1 |

[a]Lungs examined 21 days following tumour transplantation; [b]Primary tumours were treated 5 days following tumour transplantation; [c]No response – tumour did not respond to treatment; [d]Partial response – local tumour regrowth prior to sacrifice; [e]Complete response – no local tumour present at the time of sacrifice; [f]Two tailed Student *t* test: *Surgery (complete response) *vs* PDT (complete response); [g]Mean ±s.e.

colonies for mice with complete responses were similar to those shown in Table I but in this case there was no statistical significant difference between surgery and PDT treatment. No conclusion can be made for the 400 J cm$^{-2}$ PDT dose since only two mice had complete responses.

Table III shows the incidence of metastatic lung colonies in LLC bearing mice when PDT was followed by surgery. For these experiments, 200 J cm$^{-2}$ of PDT was used and this dose of PDT by itself did not induce any complete responses. The combination of PDT on day 5 and surgery on day 6 resulted in 8 mice having a partial response and 11 mice having a complete response. For mice with complete responses, the combination of PDT and surgery induced a decreased number of metastatic lung colonies compared to either surgery alone (performed on either day 5 or day 6) or Photofrin II and surgery.

Table IV documents the incidence of metastatic lung colonies in C57BL/6 mice when localized PDT preceeded LLC transplantation. PDT delivered 24 h prior to LLC transplantation had no effect on subsequent induction of metastatic lung colonies. Light doses ranged from 100–400 J cm$^{-2}$ and the left hind limb was used for PDT exposure while the right hind limb was used for tumour cell transplantation. Contralateral legs were used for this set of

**Table III** Effect of photodynamic therapy followed by surgery on the incidence of lung metastasis in C57BL/6 mice with Lewis lung carcinoma[a].

| | | *Average number of lung colonies* | | | |
|---|---|---|---|---|---|
| *Treatment* | *Number of mice* | *No response*[b] | *Partial response*[c] | *Complete response*[d] | *P values*[e] |
| Control | 83 | 67.9±8.5 | | | |
| Photofrin II (10 mg kg$^{-1}$) | 33 | 64.0±6.3 | | | |
| Surgery (day 5) | 22 | | | 18.5±6.6 | |
| Surgery (day 5) | 10 | | 13.9± 6.1 | | |
| Surgery (day 6) | 31 | | | 29.9±4.0 | |
| Photofrin II (day 4) plus Surgery (day 6) | 11 | | | 43.5±9.7 | |
| PDT (day 5)[f] | 7 | | 34.1±11.4 | | |
| PDT (day 5) plus Surgery (day 6) | 8 | | 42.6±12.4 | | |
| PDT (day 5) plus Surgery (day 6) | 11 | | | 6.6±3.6 | >0.1* <0.01† |

[a]Lungs examined 21 days following tumour transplantation; [b]No response – tumour did not respond to treatment; [c]Partial response – local tumour regrowth prior to sacrifice; [d]Complete response – no local tumour present at time of sacrifice; [e]Two tailed Student *t* test: *Surgery (day 5) *vs* PDT (day 5)+surgery (day 6), †Surgery (day 6) *vs* PDT (day 5) +surgery (day 6); [f]Photodynamic therapy (PDT) consisted of a 10 mg kg$^{-1}$ dose of Photofrin II followed 24 h later by 200 J cm$^{-2}$ delivered at 150 mW cm$^{-2}$.

**Table IV** Incidence of lung metastasis in C57BL/6 mice when photodynamic therapy preceeds Lewis lung carcinoma transplantation[a,b].

| *Treatment* | *Number of mice* | *Average number of lung colonies* | *P values*[c] |
|---|---|---|---|
| Control | 15 | 72.1± 9.6[d] | |
| Photofrin II | 11 | 86.3± 8.2 | >0.1 |
| PDT, 100 J cm$^{-2}$ | 10 | 95.3±15.3 | >0.1 |
| PDT, 200 J cm$^{-2}$ | 12 | 76.6±13.3 | >0.1 |
| PDT, 300 J cm$^{-2}$ | 10 | 72.3± 7.5 | >0.1 |
| PDT, 400 J cm$^{-2}$ | 9 | 73.0± 8.5 | >0.1 |

[a]Lungs examined 21 days following tumour transplantation; [b]Photofrin II (10 mg kg$^{-1}$) administered 48 h prior to transplantation, PDT was delivered to 1 cm diameter area of the left hind leg 24 h prior to tumour transplantation to the right hind leg; [c]Two tailed Student *t* test; [d]Mean ±s.e.

experiments in order to avoid possible artifacts related to tumour bed effects.

### Discussion

There is no evidence from the limited number of clinical PDT studies that this modality induces an increase in the metastatic rate or potential of malignant tumours. However, there are similarities between PDT and hyperthermia in that both procedures can induce significant damage to the tumour vasculature (Star *et al.*, 1986; Berenbaum *et al.*, 1986; Hahn, 1982; Eddy, 1980). Direct trauma or damage to a primary tumour may cause an increase in the number of tumour cells released into the circulation (Hill & Denekamp, 1982), although this process may not always induce metastasis (Salisbury, 1975). In the case of hyperthermia, there still remains a controversy related to its role in the spread of metastasis (Hahn, 1982; Hill & Denekamp, 1982; Oda *et al.*, 1985; Yerulshalmi, 1970; Hahn *et al.*, 1979). The majority of evidence suggests that localized hyperthermia does not effect metastatic spread, whereas total body hyperthermia appears to increase the incidence of metastatic spread. Stress induced by surgery is also suggested to play a role in a hyper-metastatic state in the *in vivo* LLC tumour model (Pollak *et al.*, 1984). The results of our study indicate that localized PDT does not enhance the dissemination of tumour metastasis in the LLC tumour model. Mice treated with PDT had significantly lower numbers of metastatic lung colonies than comparable control groups. In fact, the number of metastatic lung colonies observed following a range of PDT doses which produced either partial or complete local tumour responses were either comparable to or lower than that observed in mice treated by local surgical excision of the tumour. Treatment of tumour-bearing mice with Photofrin II alone or light treatment alone, as well as PDT delivered to mice prior to LLC transplantation had no effect on local tumour growth or subsequent lung metastasis. While extrapolation of *in vivo* data to possible clinical effects is not possible, it would appear that PDT will not induce significant side-effects related to metastatic spread of malignant tumours.

Mice with subcutaneously growing LLC develop pulmonary metastases in a reproducible manner. In addition, the LLC tumour has not been observed to regress spontaneously (Yerushalmi, 1976), and has been reported to be responsive to local PDT treatment (Cowled & Forbes, 1985). The LLC tumour model has also been used to study metastatic spread following local and whole body hyperthermia (Oda *et al.*, 1986; Yerushalmi, 1976), surgery (Pollack *et al.*, 1984), prostaglandins (Young & Knies, 1984), cytotoxic drugs (Stahl *et al.*, 1985) and anaesthetics (Shapiro

*et al.*, 1981). Our study was designed to utilize the LLC tumour model to specifically examine the role of localized PDT in the induction of metastases. It is of interest to note that relatively high doses of PDT were required for local tumour control during the 2 week period between treatment and sacrifice of animals. Partial responses (or tumour recurrences) developed in 13 out of 18 mice receiving a $10\,mg\,kg^{-1}$ dose of Photofrin II and a $400\,J\,cm^{-2}$ light dose (Table I). Eight out of 14 mice treated with Photofrin II and a $500\,J\,cm^{-2}$ dose of light also had recurrences within the 2 week period. The high degree of resistance of the LLC to PDT is probably not due to tumour tissue hypoxia since the LLC has been shown to be sensitive to ionizing radiation (Shipley *et al.*, 1975; Lvovsky *et al.*, 1985). Skin pigmentation and light attenuation may play a role in the *in vivo* PDT resistance observed in the LLC.

Recent studies have shown that PDT can induce systemic immunosuppression related to inhibition of contact hypersensitivity of dinitrofluorobenzene (Elmets & Bowen, 1986). The immunosuppression induced by PDT may be mediated by activation of the complement system (Lim *et al.*, 1984; Lim *et al.*, 1985). We have observed a transient decrease in splenic natural killer (NK) cell activity following localized PDT in mice (Gomer *et al.*, 1986). Therefore, while PDT does not enhance metastatic spread in an experimental animal model, it does appear as though this modality can induce immunosuppression related to both T cells (documented in hypersensitivity reactions) and NK cells. However, localized PDT delivered to mice prior to LLC transplantation did not effect either the rate or quantity of subsequent metastatic lung colonies.

In summary, the documented side-effects related to PDT continue to be restricted to transient skin photosensitization. The current study indicates that local PDT does not enhance the spread of tumour metastasis. Additional investigations related to this area would appear warranted in view of both the vascular and immunological action of PDT. However, since PDT has been shown to be an effective tumoricidal procedure, the results of this study would support the continued clinical examination of PDT in the treatment of both advanced as well as early stage malignancies.

We thank Barbara Paul for assistance in the preparation of this manuscript. This investigation was performed in conjunction with the Clayton Foundation for Research and was supported in part by USPHS Grant CA-31230 awarded by NCI, NHHS.

### References

BENSON, R.C. (1985). Treatment of diffuse transitional cell carcinoma *in-situ* by whole bladder hematoporphyrin derivative photodynamic therapy. *J. Urology*, **134**, 675.

BERENBAUM, M.C., HALL, G.W. & HOYES, A.D. (1986). Cerebral photosensitization by hematoporphyrin derivative. Evidence for an endothelial site of action. *Br. J. Cancer*, **53**, 81.

BRUCE, R.A. (1984). Evaluation of hematoporphyrin photoradiation therapy to treat choroidal melanomas. *Lasers Surg. Med.*, **4**, 59.

CHENG, M.K., McKEAN, J., BOISVERT, D. & TULIP, J. (1986). Photoradiation therapy: Current status and applications in the treatment of brain tumors. *Surg. Neurol.*, **25**, 423.

CORTESE, D.A. & KINSEY, J.H. (1984). Hematoporphyrin derivative in the treatment of bronchogenic carcinoma. *Chest*, **86**, 8.

COWLED, P.A. & FORBES, I.J. (1985). Photocytotoxicity *in-vivo* of haematoporphyrin derivative components. *Cancer Lett.*, **28**, 111.

DOIRON, D.R. & GOMER, C.J. (eds.) (1984). *Porphyrin Localization and Treatment of Tumors*. Alan R. Liss: New York.

DOIRON, D.R., KELLER, G.S., PROFIO, A.E. & FOUNTAIN, S.W. (1984). *Fiber-optic Delivery and Detection System for HpD Photodynamic Therapy*. SPIE, **494**, Novel Fiber Techniques for Medical Applications, p. 56.

DOUGHERTY, T.J. (1984). Photodynamic therapy (PDT) of malignant tumors. *CRC Crit. Rev.*, 83.

DOUGHERTY, T.J., WEISHAUPT, K.R. & BOYLE, D.G. (1982). Photoradiation therapy of malignant tumors. In *Principles and Practices of Oncology*, DeVita, V. *et al.* (eds) p. 1836. J.B. Lippincott: Philadelphia.

EDDY, H.A. (1980). Alterations in tumor microvasculature during hyperthermia. *Radiology*, **137**, 515.

ELMETS, C.A. & BOWEN, K.D. (1986). Immunological suppression in mice treated with hematoporphyrin derivative photoradiation. *Cancer Res.*, **46**, 1608.

GOMER, C.J. & DOUGHERTY, T.J. (1979). Determination of $^{3}H$ and $^{14}C$-hematoporphyrin derivative distribution in malignant and normal tissue. *Cancer Res.*, **39**, 146.

GOMER, C.J., FERRARIO, A. & TYAGI, R. (1986). Metastatic potential and natural killer cell activity in mice following porphyrin photodynamic therapy. *Proc. Am. Assoc. Cancer Res.*, **27**, 399.

GOMER, C.J., RUCKER, N., BANERJEE, A. & BENEDICT, W.F. (1983). Comparison of mutagenicity and induction of sister chromatid exchange in Chinese hamster cells exposed to hematoporphyrin derivative photoradiation, ionizing radiation or ultraviolet radiation. *Cancer Res.*, **43**, 2622.

HAHN, E.W., ALFIERI, A.A. & KIM, J.H. (1979). The significance of local tumor hyperthermia/radiation on the production of disseminated disease. *Int. J. Radiat. Oncol. Biol. Phys.*, **5**, 819.

HAHN, G.M. (1982). *Hyperthermia and Cancer*. Plenum Press: New York.

HAYATA, Y., KATA, H., KONAKA, C. *et al.* (1984). Photoradiation therapy with hematoporphyrin derivative in early state lung cancer. *Chest*, **86**, 169.

HENDERSON, B.W., WALDOW, S.M., MANG, T.S., POTTER, W.R., MALONE, P.B. & DOUGHERTY, T.J. (1985). Tumor destruction and kinetics of tumor cell death in two experimental mouse tumors following photodynamic therapy. *Cancer Res.*, **45**, 572.

HERRERA-ORNELAS, L., PETRELLI, N.J., MITTLEMAN, A., DOUGHERTY, T.J. & BOYLE, D.G. (1986). Photodynamic therapy in patients with colorectal cancer. *Cancer*, **57**, 677.

HILL, S.A. & DENEKAMP, J. (1982). Does local tumor heating in mice influence metastatic spread? *Br. J. Radiol.*, **55**, 444.

LIM, H.W., POL-FITZPATRICK, M.B. & GIGLI, I. (1984). Activation of the complement system in patients with porphyrins after irradiation *in-vivo*. *J. Clin. Invest.*, **74**, 1961.

LIM, H.W., YOUNG, L., HAGAN, M. & GIGLI, I. (1985). Delayed phase of hematoporphyrin-induced phototoxicity: Modulation by complement, leukocytes and antihistamines. *J. Invest. Derm.*, **84**, 114.

LITTLE, F.M., GOMER, C.J., HYMAN, S. & APUZZO, M. (1984). Observations in studies of quantitative kinetics of tritium labeled hematoporphyrin derivative in the normal and neoplastic rat brain model. *J. Neuro-Oncology*, **2**, 361.

LVOVSKY, E.A., MOSSMAN, K.L., LEVY, H.B. & DRITSCHILO, A. (1985). Response of mouse tumor to interferon inducer and radiation. *Int. J. Radiat. Oncol. Biol. Phys.*, **11**, 1721.

McCAUGHAN, J.S., WILLIAMS, T.E. & BETHEL, B.M. (1985). Palliation of esophageal malignancy with photodynamic therapy. *Annal. Thoracic Surg.*, **40**, 113.

MOAN, J. (1986). Porphyrin photosensitization and phototherapy (yearly review). *Photochem. Photobiol.*, **43**, 681.

MOORE, J. (1984). An optimized laser system for the evaluation of HPD therapy. In *Porphyrins in Tumor Phototherapy*, Andreoni, A & Cubeddu, R. (eds) p. 293. Plenum Press: New York.

NSEYO, U.O., DOUGHERTY, T.J. & BOYLE, D.G. (1985). Whole bladder photodynamic therapy for transitional cell carcinoma of bladder. *Urology*, **26**, 274.

ODA, M., KOGA, S. & MAETA, M. (1985). Effects of total body hyperthermia on metastases from experimental mouse tumors. *Cancer Res.*, **45**, 1532.

ODA, M., KOGA, S. & MAETA, M. (1986). Mechanism of metastatic spread by 42°C total body hyperthermia in Lewis lung carcinoma. *Cancer Res.*, **46**, 1102.

POLLACK, R.E., BABCOCK, G.F., ROMSCHAHL, M.M. & NISHIOKA, K. (1984). Surgical stress-mediated suppression of murine natural killer cell cytotoxicity. *Cancer Res.*, **44**, 3888.

SALISBURY, A.J. (1985). The significant of the circulating cancer cell. *Cancer Treatment Rev.*, **2**, 55.

SELMAN, S.H., KREIMER-BIRNBAUM, M., KLAUNIG, J.E., GOLDBLATT, P.J., KECK, R.W. & BRITTON, S.L. (1984). Blood flow in transplantable bladder tumors treated with hematoporphyrin derivative and light. *Cancer Res.*, **44**, 1924.

SHAPIRO, J., JERSKY, J., KATZAV, S., FELDMAN, M. & SEGAL, S. (1981). Anesthetic drugs accelerate the progression of postoperative metastases of mouse tumors. *J. Clin. Invest.*, **68**, 678.

SHIPLEY, Q., STANLEY, J. & STEEL, A. (1975). Tumor size dependency in the radiation response of the Lewis lung carcinoma. *Cancer Res.*, **35**, 2488.

STAHL, K.W., MATHE, G. & KOVACS, G. (1984). Decrease of metastogenic potential by pregraft treatment of Lewis lung carcinoma cells with proteinase and protein kinase affinity labels. *Cancer Res.*, **40**, 5335.

STAR, W.M., MARIJNISSEN, J.P.A., VAN DEN BERG-BLOK, A., VERSTEEG, A.C., FRANKEN, K.A.P. & REINHOLD, H.S. (1986). Destruction of rat mammary tumor and normal tissue microcirculation by hematoporphyrin derivative photoradiation observed *in-vivo* in sandwich observation chambers. *Cancer Res.*, **46**, 2532.

TSE, D.T., KERSTEN, R.C. & ANDERSON, R.L. (1984). Hematoporphyrin derivative photoradiation therapy in managing nevoid basal-cell carcinoma syndrome. *Arch. Ophthalmol.*, **102**, 990.